# Fundus Autofluorescence and RPE Lipofuscin in Age-Related Macular Degeneration

**DOI:** 10.3390/jcm3041302

**Published:** 2014-11-17

**Authors:** Janet R. Sparrow, Tobias Duncker

**Affiliations:** 1 Department of Ophthalmology, Columbia University Medical Center, 635 W. 165th Street, New York, NY 10032, USA; E-Mail: tobias.duncker@gmail.com; 2 Department of Pathology and Cell Biology, Columbia University Medical Center, 630 168th Street, New York, NY 10032, USA

**Keywords:** retinal pigment epithelium, bisretinoid, melanin, lipofuscin, fundus autofluorescence, near-infrared fluorescence, short-wavelength fluorescence imaging

## Abstract

Genes that increase susceptibility to age-related macular degeneration (AMD) have been identified; however, since many individuals carrying these risk alleles do not develop disease, other contributors are involved. One additional factor, long implicated in the pathogenesis of AMD, is the lipofuscin of retinal pigment epithelium (RPE). The fluorophores that constitute RPE lipofuscin also serve as a source of autofluorescence (AF) that can be imaged by confocal laser ophthalmoscopy. The AF originating from lipofuscin is excited by the delivery of short wavelength (SW) light. A second autofluorescence is emitted from the melanin of RPE (and choroid) upon near-infrared (NIR-AF) excitation. SW-AF imaging is currently used in the clinical management of retinal disorders and the advantages of NIR-AF are increasingly recognized. Here we visit the damaging properties of RPE lipofuscin that could be significant when expressed on a background of genetic susceptibility. To advance interpretations of disease-related patterns of fundus AF in AMD, we also consider the photochemical and spectrophotometric features of the lipofuscin compounds responsible for generating the fluorescence emission.

## 1. Introduction

Age-related macular degeneration (AMD) is a complex disorder that is influenced by genetic and environmental factors; heritability is estimated to account for 45% to 71% of cases [1,2]. Several (~20) AMD genetic susceptibility genes have been identified with two loci, complement factor H (CFH (1q32) and age-related maculopathy susceptibility 2/HtrA serine peptidase 1 (ARMS2/HTRA1) on 10q26 accounting for 50% of AMD cases [3,4]. The CFH locus harbors many independent risk and protective haplotypes [5,6] while for ARMS2/HTRA1 a single major risk haplotype is associated with AMD [7]. Multiple studies have also demonstrated that haplotype-tagging single-nucleotide polymorphisms (SNPs) in CFH and ARMS2, are major determinants of AMD endophenotypes and disease progression [8]. Specifically, CFH-rs1061170 is associated with drusen and with both early and advanced AMD while ARMS2-rs10490924 is strongly associated with reticular pseudodrusen and rapid progression to late AMD [5,7,8,9]. Nevertheless, by various estimates, currently known genetic loci account for only 50%–75% of overall AMD risk. Interest in a role for retinal pigment epithelium (RPE) lipofuscin in AMD stems from the knowledge that it accumulates with age [10,11], is high in central retina [11], exhibits behaviors toxic to RPE [12,13,14,15] and exhibits a link to drusen formation [16]. 

## 2. RPE Lipofuscin as the Source of Short-Wavelength (SW)-Fundus Autofluorescence

Advances in non-invasive fundus imaging have facilitated the diagnosis and differentiation of retinal disease. *In vivo* imaging provides a window within which to view the natural course of retinal disease. Of the available imaging modalities, fundus autofluorescence (AF) has proven to be especially valuable, in large part because disease-related processes can alter the distribution of the AF signal. Accordingly, recognizable disease phenotypes are often produced. 

**Figure 1:**
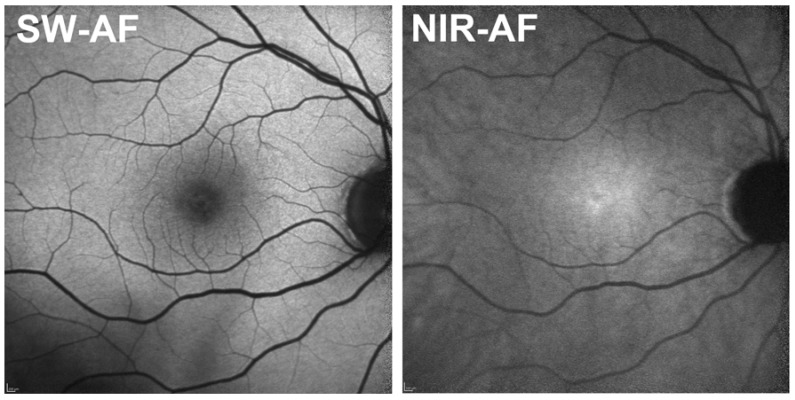
Short-wavelength (SW-AF) and near-infrared (NIR-AF) fundus autofluorescence. Images were obtained with 488 nm (SW) and 787 nm (NIR) excitation.

The natural autofluorescence of the fundus that is excited by SW light (488 nm excitation) (Figure 1) exhibits spectral features and an age-relationship that indicates a principle origin from the fluorescent pigments that accumulate in RPE cells as lipofuscin [17]. Unlike lipofuscin species that accumulate in other non-dividing cells, the pigments of RPE lipofuscin are produced in the membranes of photoreceptor outer segments from non-enzymatic reactions of vitamin A aldehyde [18,19,20,21]. This fluorescent material is transferred to RPE cells within phagocytosed outer segment disks [22,23] and becomes deposited in the lysosomal compartment of the cells. In the healthy retina, fundus autofluorescence increases linearly with age although subjects vary in terms of intensities [11]. The age-related increase levels off after age 70 perhaps because of a loss of photoreceptor or RPE cells [24] and/or changes in fluorescence emission due to extensive photooxidation/photodegradation of the bisretinoid compounds [25] (discussed below). 

**Figure 2:**
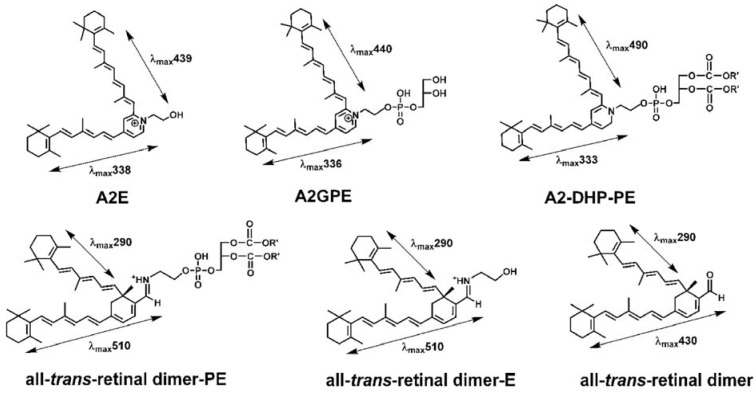
Structures and absorbance maxima (λ_max_) of some bisretinoid fluorophores in retinal pigment epithelium (RPE) lipofuscin. Absorbance maxima can be assigned to each of the side-arms of the molecules.

RPE lipofuscin consists of a complex mixture of fluorophores that have been identified in by chromatography and mass spectrometry and characterized structurally; all of the known bisretinoid lipofuscin pigments have been detected in human eyes [26] (Figure 2). These fluorophores include the pyridinium-containing molecules A2-glycerophosphoethanolamine (A2-GPE) [27], A2E and isomers of A2E [28,29,30,31,32,33,34,35,36], dimers of all-*trans*-retinal having a cyclohexadiene head group (all-*trans*-retinal dimer) [33,37] and the associated protonated Schiff base conjugate [37] and the uncharged A2-DHP-PE (A2-dihydropyridine-phosphatidylethanolamine) [38]. Higher molecular weight adducts also form when aldehyde-bearing cleavage products of bisretinoid react with intact bisretinoid molecules [39]. Other molecular constituents of RPE lipofuscin are adducts of CEP (2-(ω-carboxyethyl)-pyrrole) [40], HNE (4-hydroxynonenal) and MDA (malondialdehyde) [41] that are derived from oxidative fragmentation of lipid. Products of lipid oxidation are generally non-fluorescent or blue-emitting fluorophores [42,43] and in this case could be generated by the photoreactivity of other lipofuscin fluorophores. Little or no protein is present in RPE lipofuscin [40]. Accumulation of bisretinoids in RPE cells is unlikely to depend on an inhibition of lysosomal enzyme activity, since this fluorescent material is amassed in all healthy eyes beginning at early ages [44].

The various bisretinoids of RPE lipofuscin have some common structural features (Figure 2). They present with a central six-carbon ring from which extend two polyene arms terminating in β ionone rings. Each of these arms is derived from a molecule of retinaldehyde and constitutes a separate light-absorbing chromophore, one arm absorbing in the ultraviolet range and the other in the visible (Figure 2). The numbers of alternating carbon-carbon double and single bonds that form the conjugation systems of the arms determine the wavelength of absorbance; the longer conjugation system in each molecule confers absorbance in the visible range. Absorbances in the visible spectrum are significant since these wavelengths reach the retina. Since these adducts of retinaldehyde are held together by covalent bonds, bisretinoids do not provide stores of retinoid for the visual cycle, as has been suggested [45].

## 3. Spectral Signatures of SW-Fundus AF and RPE Lipofuscin

**Figure 3:**
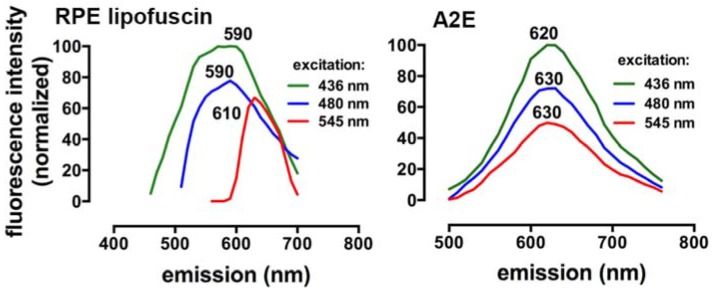
Fluorescence emission spectra of human RPE lipofuscin and A2E (in PBS with 2% DMSO). Emission was recorded at excitation wavelengths 436, 480 and 545 nm as published [49]. Emission maxima are indicated.

In clinical settings, SW-fundus AF is excited by wavelengths ranging from 488 nm, the excitation employed with a confocal scanning laser ophthalmoscope (cSLO), to the 535–580 nm range utilized by a modified fundus camera [46] and the 568 nm light used with fluorescence adaptive optics ophthalmoscopy [17,47,48]. Fundus autofluorescence measured *in vivo* by spectrophotometry has a broad excitation spectrum that peaks between 490–510 nm. The fluorescence emission is also broad and centered at approximately 600 nm [11,17]. RPE lipofuscin *ex vivo* exhibits an excitation spectrum that peaks between 450–490 nm; the fluorescence emission is maximal at ~600 nm [49] (Figure 3). Moreover, just as with fundus autofluorescence, the emission spectrum recorded from whole lipofuscin exhibits red-shifts when excited by progressively longer wavelengths [49] (Figure 3). Thus the spectral characteristics of fundus autofluorescence are consistent with that of RPE lipofuscin [43,50,51,52] and chiefly with an origin from the bisretinoid fluorescent pigments that are known constituents of RPE lipofuscin. The bisretinoids that have been characterized have absorbance maxima varying from 440 nm to 510 nm and they emit with an orange fluorescence that peaks at ~600 nm [26]. The bisretinoid A2E can emit fluorescence at longer wavelength excitations such as 545 nm (Figure 3B).

## 4. Photoreactive Properties of RPE Lipofuscin and the Implications for Fundus AF 

While there exists no evidence that bisretinoids of RPE lipofuscin can undergo lysosomal degradation, loss of this material due to photodegradation has been demonstrated. Thus studies of RPE lipofuscin [53,54,55] and individual bisretinoid lipofuscin fluorophores such as A2-GPE, all-*trans*-retinal dimer and A2E have revealed that these compounds are photoinducible generators of reactive oxygen species such as singlet oxygen and superoxide anion. Singlet oxygen in turn reacts with the conjugated double bond systems comprising the arms of the bisretinoid molecules [56,57,58,59,60,61] leading to the fragmentation of the parent molecules and release of aldehyde-bearing cleavage products such as methylgloxal and glyoxal [60] that can react with and inactivate proteins. These small dicarbonyls also provoke the formation of advanced glycation end products (AGE) that deposit extracellularly [16,62]. 

AGEs incite inflammatory processes and since they are detected in drusen [63,64], they reflect a link between RPE bisretinoid lipofuscin and the formation of sub-RPE deposits. Photooxidation of A2E and all-*trans*-retinal dimer has also been shown to incite complement activation [65,66].

Photooxidation is clearly an ongoing process in the eye since photooxidized forms of A2E and all-t*rans*-retinal dimer have been detected in isolated human and mouse RPE [25,57]. These processes likely contribute to Bruch’s membrane thickening [67] and photoreceptor cell degeneration [68,69] in Abca4 mutant mice and are a cause of the increased vulnerability of albino Abca4^−/−^ mice to retinal light damage [70].

**Figure 4:**
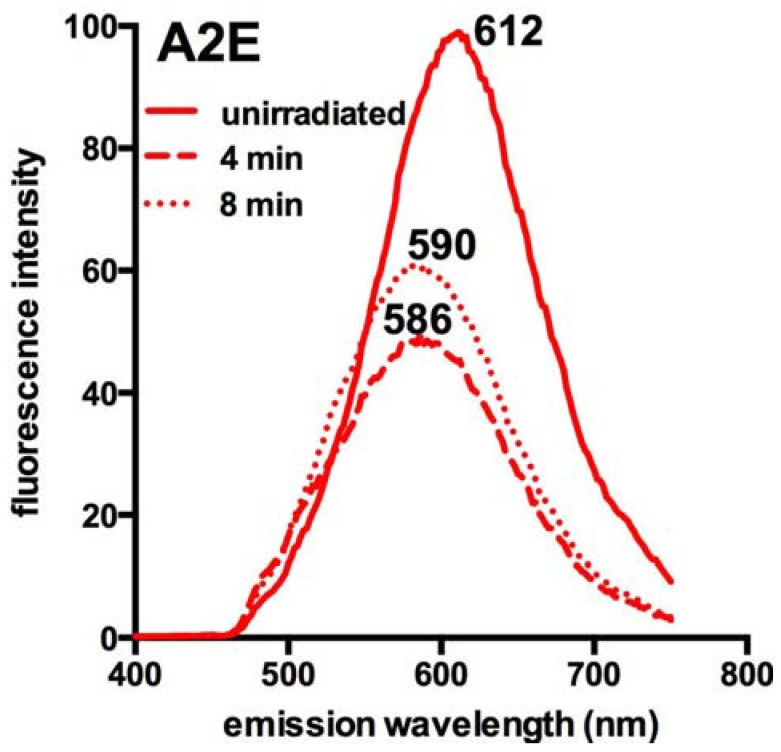
A2E photobleaching by irradiation at 480 nm for 4 and 8 min. Fluorescence intensity decreases with irradiation and the emission maximum undergoes a hypsochromic shift. Emission peak wavelengths (nm) are indicated adjacent to each trace.

**Figure 5:**
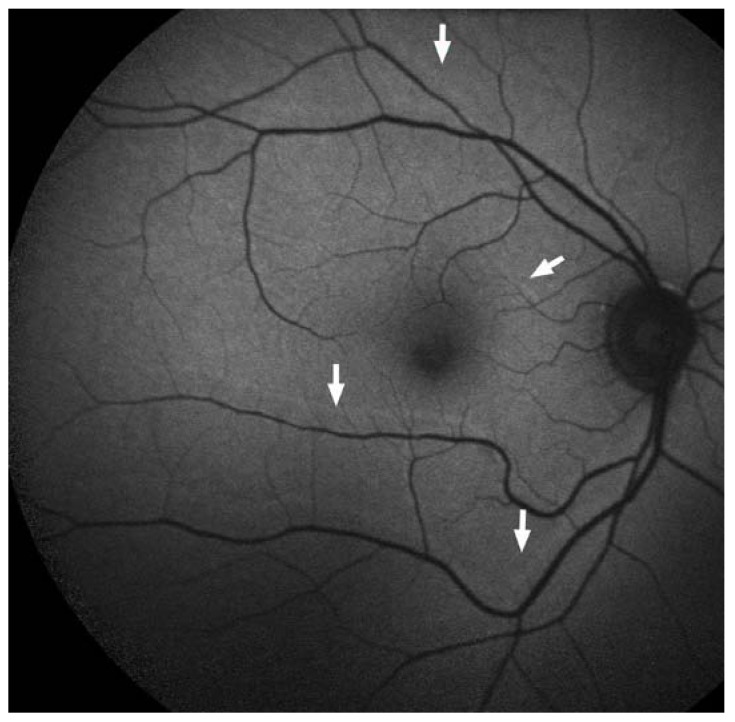
Fundus autofluorescence image of an individual following retinal detachment repair. Hyperautofluorescent lines follow a course that parallels the retinal vessels below.

The propensity for bisretinoids to undergo photooxidative and photodegradative processes may underlie the decline in RPE lipofuscin fluorescence emission (photobleaching) that has been observed in non-human primates during *in vivo* fluorescence imaging by adaptive optics scanning laser ophthalmoscopy [48,71], with cell culture models [72] and in non-cellular assays (Figure 4). Lipofuscin photobleaching may also explain why after surgical repair of some cases of retinal detachment, hyperautofluorescent lines coursing parallel to retinal blood vessels can be visible in fundus AF images [73,74] (Figure 5). The hyperautofluorescent imprint has been interpreted as indicating a change in the position of the vessel relative to the underlying retinal tissue and is visible because of contrasting levels of AF brightness. At any given time, the intensity of fundus AF is likely the difference between fluorophore synthesis on the one hand, and lipofuscin photoxidation/photodegradation in RPE, on the other. Under the shadow of a blood vessel, the formation of bisretinoid from retinaldehyde (with 11-*cis* being converted to all-*trans*-retinal) would likely continue unabated [20,30,75,76,77] but lipofuscin photooxidation and photobleaching would be substantially reduced. As a result, a vessel imprint of more intense AF would be revealed upon retinal translocation.

## 5. Topographic Distribution of SW-AF in Healthy Eyes

**Figure 6:**
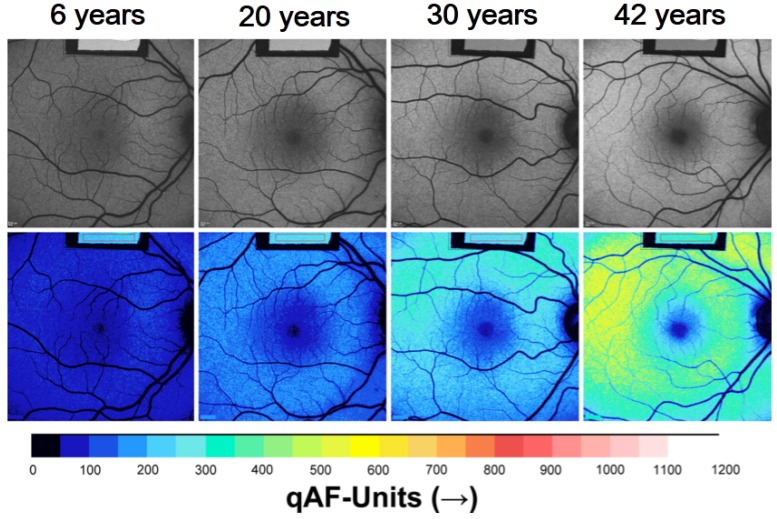
Quantitative fundus autofluorescence (qAF) in healthy human eyes. Short-wavelength fundus AF images (top row) and corresponding color-coded qAF images (bottom row) at the ages indicated. Lower qAF values are coded in blue and higher qAF values as orange (color scale). Fundus autofluorescence intensities increase with age and the highest levels occur in superior-temporal fundus.

When RPE lipofuscin is assayed by recording fluorescence in histological sections of human retina, the signal is found to increase from central fovea to perifovea and after peaking at an eccentricity of ~8°, it decreases towards the periphery [10,78]. A similar pattern has been observed with quantitative fundus autofluorescence (qAF) (Figure 6); at an eccentricity of 10°, qAF is approximately 95% of that measured centrally [79]. Reduced foveal fundus autofluorescence is due in large part to absorption of the exciting light by macular pigment and to the higher optical density of melanin in central RPE [17]. By fundus spectrophotometry, qAF and fluorescence photomicroscopy [45], the highest levels of RPE lipofucin in healthy eyes have been observed perifoveally in superior-temporal retina (Figure 6) [44]. Unexpectedly, this pattern was not replicated in another study relying on fluorescence measurements in flat-mounted human cadaver eyes [80]. 

Based on the spatial distribution of mass peaks detected with matrix-assisted laser desorption-ionization imaging mass spectrometry (MALDI-MS) investigators have reported that A2E is detectable in central retina of mice [81], but not in the central human RPE [36]. Instead, the highest A2E levels were restricted to a small patch of RPE found exclusively in the far periphery of temporal retina [80]. This patch was not matched by similar A2E signal in in the periphery of superior, nasal or inferior retina. While A2E is well known to be fluorescent, surprisingly, this patch was almost devoid of fluorescence.

Since the spectral features of RPE lipofuscin and fundus autofluorescence are best accounted for by the excitation and emission spectra of the bisretinoid constituents, the fluorescence originating in centrally situated RPE cells likely originates from some combination of these di-retinal adducts. Interestingly, the MALDI-MS findings could indicate that the various species of bisretinoid lipofuscin compounds exhibit spatial heterogeneity. Reduced levels of A2E in the macula could also be a consequence of greater lipofuscin photocleavage in central RPE; an explanation such as this could account for the greater susceptibility of the macula to disease. Failure to detect A2E centrally, may even be attributable to the limitations of the methodology. MALDI-MS is a surface-based technique that depends on the extraction of analyte into a matrix applied to the surface of the tissue. Factors that could cause spatial differences in extraction efficiency are regional differences in RPE height and spatial differences in the compartmentalization of lipofuscin. For instance RPE cells are taller and narrower in the human macula [78] with melanin being uppermost and lipofuscin at greater depths in the cells. After age 50 complex organelles containing both melanin and lipofuscin (melanolipofuscin) predominate in the cells [78] with macular RPE containing more melanolipofuscin than RPE at the equator and periphery [82]. Lipofuscin is less extractable from the RPE of these older eyes [83]. Thus it is likely that the extraction from central RPE is less efficient because of the depths of lipofuscin in the cells and/or difficulty in accessing lipofuscin from complex melanolipofuscin-organelles.

## 6. NIR-AF in the Healthy Eye

The healthy fundus also exhibits a near-infrared autofluorescence (NIR-AF) (>800 nm) when excited at ~787 nm [84,85] (Figure 1). The intensity of the fundus NIR-AF is at least 60 times less than SW-AF [86]. Several lines of evidence indicate that RPE and choroidal melanin serve as a source of the NIR-AF signal. For instance, melanin is known to fluoresce under near-infrared light excitation [87]. Additionally, the high NIR-AF signal at the fovea corresponds [86] to the elevated optical density of melanin in this area [78]. Melanocytic choroidal nevi also fluoresce brightly with NIR-AF imaging. Conversely, NIR-AF emanating from a full-thickness macular hole is similar in brightness to surrounding retina [86]. 

The more frequent use of SW-AF may be due, in part, to the introduction of the Heidelberg Spectralis having optical coherence tomography (OCT) capability (HRA + OCT) and to the subsequent decline in the popularity of the HRA2 and Spectralis HRA in retinal clinics. The OCT module in the Spectralis reduces NIR-AF signal intensity, thus compromising NIR-AF image quality as compared to cSLOs without OCT (e.g., HRA2 and Spectralis HRA). Nevertheless, NIR-AF has advantages over SW-AF. For instance, during image acquisition, patients are not disturbed by the NIR-AF light as they are with the SW-AF exciting light. This improves patient cooperation, especially in young children and photophobic patients. 

## 7. Comparison of SW- and NIR-AF in AMD: Altered Intensities and Aberrant Patterns 

**Figure 7:**
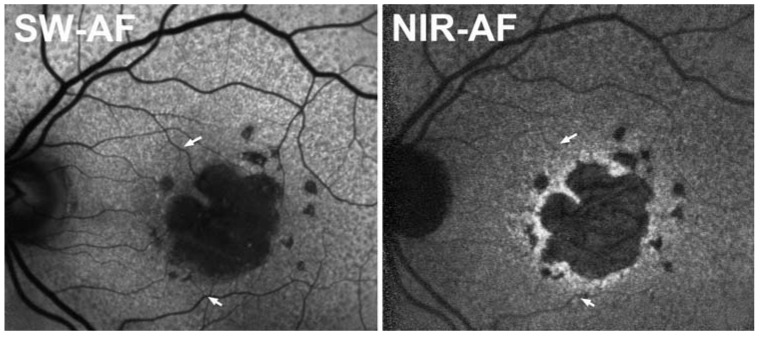
An area of geographic atrophy (GA) imaged with short-wavelength (SW) and near-infrared (NIR) autofluorescence (AF) imaging. GA appears dark with both modalities. The zone surrounding GA is hyperautofluorescent in the NIR-AF image while having relatively normal SW-AF signal. When vessel landmarks are used as a guide (arrows), total lesion size appears larger in the NIR-AF image.

In the presence of retinal disease such as AMD, patterns and intensities of fundus autofluorescence are notably altered [88,89,90,91,92,93]. At locations of RPE and photoreceptor cell demise (atrophy) both the SW-AF and NIR-AF signals become strikingly deficient or absent [94,95]. These areas of atrophy can take the form of discrete spots, isolated patches or large expanses (geographic atrophy, GA, USA). Loss of the RPE cell monolayer in GA has been confirmed by OCT [96]. While GA presents as areas of darkness in both SW and NIR-AF (Figure 7), the lesion size can sometimes appear larger with either the SW-AF or NIR-AF modality [84,97] (Figure 7). Nevertheless, the rate of increase in GA area is the same whether measured in SW- or NIR-AF images [98]. In exudative AMD, the developing neovascular lesion can be discerned in SW-AF images early on as a focal hyperautofluorescence [99]. Later the SW-AF signal is reduced within the lesion [99,100]. In some cases the edge of the neovascular lesion exhibits increased SW-AF signal [100].

In the zone of retina immediately adjacent to geographic atrophy, there are often additional AF changes. These aberrant signals can present as intermittent foci or continuous bands of altered brightness in both SW-AF and NIR-AF images [101,102,103]. In these junctional zones areas of increased SW-AF can coincide with increased NIR-AF; increased SW-AF can overlap with reduced NIR-AF; or NIR-AF signal can be enhanced while SW-AF may appear normal [84,104] (Figure 7). Interestingly, a loss of photoreceptor function was found to be associated with both increased and decreased NIR-AF [97,105]. 

Since both SW-AF and NIR-AF are considered to originate in RPE cells, it is puzzling that SW-AF can be increased at positions where NIR-AF is reduced or absent. This apparent incongruity has been attributed to abnormal RPE cells that have lost melanin while accumulating excessive levels of lipofuscin due to accelerated rates of outer segment phagocytosis [84,105]. However, lipofuscin production is not dependent on the rate of phagocytosis. The lipofuscin forms in photoreceptor cells prior to disc shedding and phagocytosis. As is evident from studies of the RCS rat, the fluorophores of lipofuscin form and accumulate in photoreceptor outer segment debris even in the absence of phagocytosis [106,107,108]. 

Other observations indicate that photoreceptor cells are likely to be degenerating in these junctional zones of increased SW-AF. In particular, retinal sensitivity, measured by microperimetry, is commonly reduced at positions presenting with increased SW-AF as compared to normal SW-AF [105,109]. In addition, OCT findings in the zone of enhanced autofluorescence surrounding GA include thinning of the reflective band attributable to RPE/Bruch’s membrane and disruption of the reflectivity band corresponding to the ellipsoid zone of photoreceptor inner segments [96,110] Thus could it be that at positions of diminished NIR-AF, RPE cells are atrophied or lost and impaired photoreceptor cells become a source of accelerated lipofuscin formation and thus enhanced SW-AF? Mechanistically, mishandling of retinaldehyde, the precursor of lipofuscin, is known to lead to elevated bisretinoid formation in photoreceptor cells [111] and compromised photoreceptor cells may not be able to provide the energy needed for reduction of retinaldehyde to the non-reactive alcohol form. Similar mechanisms may explain the rapid onset of elevated fundus SW-AF that co-localizes with scotomas associated with acute macular neuroretinopathy (AMN) [93,112]. 

**Figure 8:**
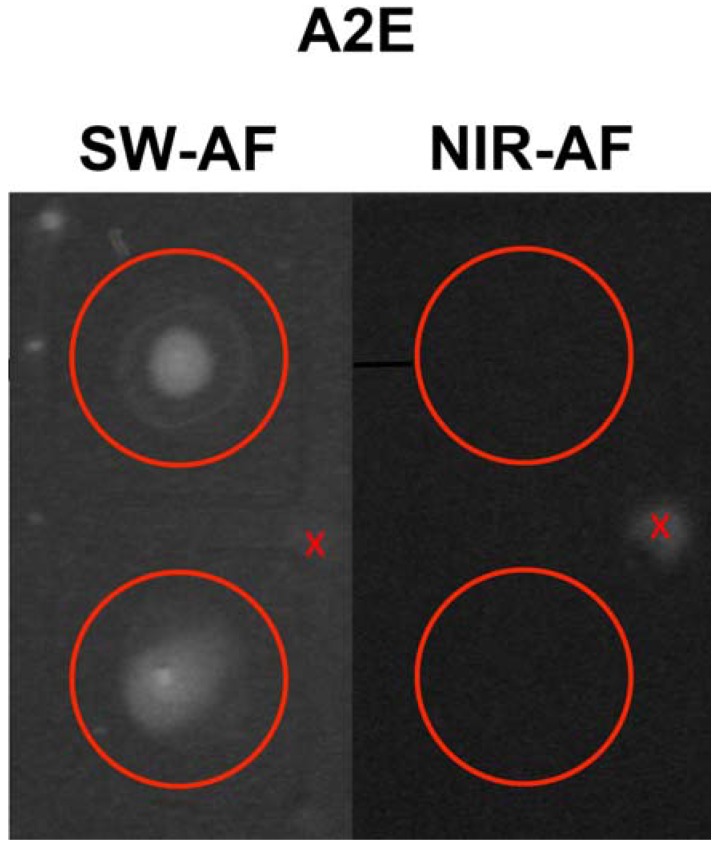
Synthesized A2E imaged with a confocal scanning laser ophthalmoscope (HRA2; Heidelberg Engineering, Heidelberg, Germany) using 488 nm (SW-AF) and 787 nm (NIR-AF) excitation. No signal was generated with the NIR excitation.

The observation that NIR-AF signals increase in parallel with increases in SW-AF at the border of GA may also not be fully understand. Hyperautofluorescence foci at the junctional zone of GA, at least in some cases, can be attributed to abnormally superimposed RPE cells [113,114]. But whether this can account for more extensive bands of high NIR-AF [84] is not certain. It has been suggested that RPE lipofuscin may contribute to NIR-AF of the fundus [86]. However, albino rats do not exhibit NIR-AF despite the presence of lipofuscin [115] and as shown in Figure 8, we have not detected an NIR-AF signal from synthesized samples of A2E that otherwise emit fluorescence when excited at 488 nm. NIR-AF emission from other bisretinoids is unlikely, given the small structural differences amongst these compounds (Figure 2). If melanogenesis is the basis for increased NIR-AF brightness at the border of GA, as has been suggested [84,105], one might expect that increased melanin concentrations would be visible as hyperpigmented spots and bands in color fundus photographs. In considering an optical effect as an explanation for increased NIR-AF signal it could be that RPE lipofuscin does not fluoresce in the NIR range but can modify the NIR-AF emission from melanin. This could occur if lipofuscin secondary lysosomes intercalate amongst apically situated melanosomes [78,116] thereby reducing the NIR-AF quenching associated with secondary self-absorbance of the fluorescence emission. A mechanism such as this might also explain why hyperautofluorescent rings visible in the NIR-AF fundus images in retinitis pigmentosa exhibit spatial correspondence with high intensity rings observed in SW-AF images [117].

## 8. Conclusions

Interest in a role for RPE lipofuscin in AMD stems from its age-related increase [10,11], an accumulation that is more pronounced in central retina [11], a propensity for adverse effects on RPE and photoreceptor cells [12,13,14,15,68,70] and links to drusen formation [16,67]. Contributions to AMD susceptibility from RPE lipofuscin would exist within the context of background genetic risk. Thus extrapolation from aging eyes in the absence of disease may not be informative [45]. 

Since the products of bisretinoid photodegradation can be damaging [16,60], it is worth considering whether lipofuscin lost by photooxidation/photodegradation is more significant than the lipofuscin remaining in the cells. At any given time, the lipofuscin in RPE that is recorded by SW-AF may consist of only some portion of the fluorescent material that has been accumulated over a life-time. SW-AF emitted from RPE bisretinoids is commonly regarded as a way to monitor the health of the RPE, with areas of high AF indicating increased lipofuscin levels and areas of low AF indicating RPE loss. However, as discussed here there is increasing evidence that interpretations of the SW-AF signal are complex. For instance, impaired photoreceptors may generate increased levels of bisretinoid fluorophores thus amplifying SW-AF signal. Ultimately, an understanding of patterns of fundus AF will impact the use of these images to assess therapeutic outcomes.

While fundus autofluorescence provides *en face* spatial information, the spectral features of the fluorescence are not elucidated and the cellular origin of the fluorescence is not identified. Recently, however, methods have been developed for quantifying SW-AF. This approach has been shown to enable the differentiation of similar phenotypes having disparate genetic origins [44,118]. The qAF approach may eventually help to ascertain the role of lipofuscin in various retinal disorders including AMD. Because of more limited gradations of signal and thus greater contrast, diseased *versus* non-diseased areas of retina are easier to distinguish in NIR-AF images as compared to SW-AF images. Indeed, in recessive Stargardt disease (STGD1), SW-AF changes are often not obvious at fundus locations where abnormalities are detectable in NIR-AF images [119]. In addition, in many cases the low NIR-AF signal corresponds spatially to loss of the inner segment ellipsoid zone (EZ) in spectral domain (SD) OCT images [119]. This relationship is important since EZ integrity is essential for visual function [120]. While the origin of the NIR-AF signal may not be completely understood, the NIR-AF signal can provide a good estimate of the size of geographic atrophy and surrounding abnormalities. All of these issues favor the inclusion of NIR-AF in the management of retinal diseases such as AMD.
